# Consumption in the Navy

**Published:** 1902-10-18

**Authors:** 


### Consumption in the Navy.

The prevalence of consumption in the navy is a matter of much public concern. True, the proportion of deaths from this disease among our sailors is considerably less than amongst the general population, say, of London; but if we remember that our blue-jackets are selected men, all of whom have passed a most careful medical examination before admission to the service, and that they are of an age at which consumption ought but seldom to arise, we need hardly hesitate to assert that the existing mortality and invaliding rate from this cause is such as ought not to exist, and ought no longer to be accepted as inevitable. Many causes have been put forward to explain the fact that, notwithstanding the apparent healthiness of a sailor's life, so many of those who serve on board our men-of-war become affected with tuberculosis. It has been asserted that in their watches they are exposed to all sorts of weather, that when below they are often crowded into a small space, that in modern battleships, where everything is built of steel and iron, the ships' sides often run down with damp from condensed moisture, that the men's quarters are either very hot or very cold, that there is no ventilation except what is forced, and lastly that such ventilation as there is is insufficient to ensure purity of air. As to all of these matters very contradictory statements have from time to time been made. It has, however, to be admitted that no one of these alleged defects would of itself set up tuberculosis, and that we must look further if we are to appreciate correctly the origin of the disease and the means to be adopted for its prevention. Although a modern man of war is almost as full of machinery as a mill it has to be dealt with somewhat differently from a factory. In any ordinary industry in which an excessive prevalence of phthisis prevailed the first thing to do would be to remove the conditions which predispose to the disease. Hence the strict provisions made by Factory Acts in regard to many sanitary matters, by compliance with which, and especially by free ventilation and the admission of light and air to every part, factories may in some instances be rendered so healthy, and so little likely to facilitate the transference of the infection from case to case, that, with due precautions against spitting, one may admit consumptive workers to them without serious risk of spreading the disease. But a man of war is first and above all things a fighting machine. Its main object is paramount in every detail of its construction, and no "fanciful" question about cubic space, or ventilation, or comfort, is allowed to stand before perfection in its warlike mission. Under these circumstances it must happen, as it undoubtedly has happened, that these fighting machines are in many ways not altogether the most delightful abodes, and that the conditions in which their crews exist are not the best imaginable for preventing the spread of any infection which may find place among them. The two main faults from a sanitary point of view are the overcrowding and the lack of ventilation, two faults which hang together, for when the men are below, so tightly are they packed in some vessels that it would require a veritable tornado of ventilation to secure anything like purity of air.

All the more necessary is it then that infection should not be allowed to gain admission, and here we have what seems to us the key to a large proportion at least of the difficulty. One cannot but be struck by the fact that far more cases of consumption are returned than are invalided, which makes it evident that cases are reported again and again, are patched up and are kept in the service for some time before they finally pass out of it dead or invalided, and that during all this time they are serving as centres of infection and spreading disease among their companions.

It is easy to see what leads to this condition of affairs. A thoroughly-trained seaman is a valuable public servant with whom no one wishes to part, especially as—at any rate during peace time—he may be quite efficient, notwithstanding his having a little hole in his lung. Moreover, to discharge such a man would often be a great hardship, meaning as it must do in many cases that he would be discharged to poverty. Hence there are many influences which make for the retention in the service of these slightly tuberculous yet still efficient sailors. But one also sees that so long as the necessities of naval construction require that sailors shall be cooped up as they are under present conditions the retention of tuberculous men until such time as they become inefficient must be an abiding cause of tuberculosis in the Navy. If we wish our gardens to be free from thistles we must knock off their heads before they cast their seed, and if we wish to keep our Navy free from phthisis we must weed out each tuberculous patient as soon as ever it is possible to identify his malady.

